# RAB18, a protein associated with Warburg Micro syndrome, controls neuronal migration in the developing cerebral cortex

**DOI:** 10.1186/s13041-016-0198-2

**Published:** 2016-02-16

**Authors:** Qinwei Wu, Xiaqin Sun, Weihua Yue, Tianlan Lu, Yanyan Ruan, Tianda Chen, Dai Zhang

**Affiliations:** Peking-Tsinghua Center for Life Sciences, Peking University, Beijing, 100871 China; Academy for Advanced Interdisciplinary Studies, Peking University, Beijing, 100871 China; Peking University Sixth Hospital/Institute of Mental Health, Beijing, 100191 China; National Clinical Research Center for Mental Disorders/Key Laboratory for Mental Health, Ministry of Health (Peking University), Beijing, 100191 China; PKU-IDG/McGovern Institute for Brain Research, Peking University, Beijing, 100871 China

**Keywords:** RAB18, Neuronal migration, Warburg Micro syndrome

## Abstract

**Background:**

Loss of function mutations in RAB18, has been identified in patients with the human neurological and developmental disorder Warburg Micro syndrome. However, the function of RAB18 in brain remains unknown.

**Results:**

In this study, we report that RAB18 is a critical regulator of neuronal migration and morphogenesis. Using in utero electroporation suppression of RAB18 in the mouse brain impairs radial migration. Overexpression of dominant negative RAB18 or disruption of RAB3GAP (RAB18GEF) also results in delayed neuronal migration in the developing mouse cortex and inhibition of neurite growth in vitro. Moreover, loss of RAB18 induces an acceleration of N-cadherin degradation by lysosomal pathway resulting in the decrease of surface level of N-cadherin on neurons.

**Conclusions:**

RAB18 regulates neuronal migration and morphogenesis during development. Our findings highlight the critical role of RAB3GAP-RAB18 pathway in the developing cerebral cortex and might explain some of clinical features observed in patients with Warburg Micro syndrome.

**Electronic supplementary material:**

The online version of this article (doi:10.1186/s13041-016-0198-2) contains supplementary material, which is available to authorized users.

## Background

Warburg Micro syndrome is an autosomal recessive neurological and developmental disorder [1]. Four loss of functional mutations *RAB18*, *RAB3GAP1*, *RAB3GAP2* and *TBC1D20* have been identified so far in this disorder [2–6]. Children with Warburg Micro syndrome appear to multiple specific developmental abnormalities in brain and eye development, and profound mental retardation [7].

RAB18 is a kind of small GTPases which are key regulators of membrane trafficking and are activated by guanine nucleotide exchange factors (GEFs) and inactivated by GTPase activating proteins (GAPs) [8, 9]. RAB18 has been linked to the regulation of secretory granules, ER-Golgi trafficking, lipid droplet formation, and virus infection in different types of cells [10–14]. However, no clear molecular function or localization has been defined for RAB18 in neurons during development. Recent in vivo studies suggest that some small GTPases RAB5, RAB7, RAB11 and ARF6 have roles in the migration of immature neurons during the development of cerebral cortex [15, 16]. Some studies show the evidence linking the functions of numerous RAB family proteins to axonal and dendritic development in primary neurons, indicating RAB18 may also have a major role in neurite outgrowth [17–20].

The children with Warburg Micro syndrome suffer from multiple specific developmental abnormalities in brain [3, 7]. More recently, *RAB18* knockout mouse phenotypes of the thinner corpus callosum provide evidence that RAB18 results in substantial and lasting defects of major projections of upper layer [21, 22]. RAB3GAP1 and RAB3GAP2 form a RAB3GAP complex which is a specific RAB18GEF and disease-associated point mutations in either the RAB3GAP1 or RAB3GAP2 subunits result in the loss of function of RAB18GEF [8]. Warburg Micro syndrome is a disease characterized by direct loss of RAB18 function or loss of RAB18 activation showing an important role of RAB18 in neuronal development [3, 8]. In the current study, by using of in utero electroporation, we found the in vivo role of RAB3GAP-RAB18 pathway in neuronal migration during the development of cerebral cortex. These findings contribute to the understanding of the pathology of Warburg Micro syndrome.

## Methods

### Animals

Pregnant mice used for in utero electroporation were purchased from Department of Laboratory Animal Science, Peking University Health Science Center. All animal experiments were approved by the institutional committee of Peking University and conducted according to the guidelines from the Ethics Committees of Peking University Health Science Center.

### Plasmid constructions

The shRNA target sequences for RAB18 and RAB3GAP2 are as follow: shRAB18-1: 5'-GCTTGTTGAGAAGATCATTCA-3', shRAB18-2: 5'-GGTGCACAGGGAGTTATATTA-3', shRAB18-3: 5'-GGATGGAAATAAGGCTAAACT-3', shRAB3GAP2-1: 5'-AGACAACAGTTCTTACTGA-3' [8], shRAB3GAP2-2: 5'-GGAATACGTTCTTGGTGAA-3' [8]. The shRNAs were cloned into SUPER vector. Expression constructs containing mouse RAB18, RAB3GAP2, and CDH2 were generated by PCR and were inserted the corresponding coding region into the pCAGGS-IRES-EGFP vector. Indicated mutants of RAB18 (S22N) and the shRAB18-3 resistant form RAB18 (RAB18^R^) were generated using the PCR based method of gene splicing by overlap extension.

### Real-time PCR

The total mRNAs were extracted from the neocortex of E14.5, E17.5, and P0 mice using TRIzol reagent (Invitrogen). Toltal cDNA was synthesized using a cDNA synthesis kit, Super-Script II reverse transcriptase (Invitrogen). Real-time PCR was performed with the KAPA SYBR FAST qPCR Kits (Kapa Biosystems) and on a Roche LC96 apparatus. The primers used to amplify the RAB18 cDNA were 5'-GGAAAACCGTGAAGTCGATAG-3' and 5'-AAGGCACACTGTACACCATCAC-3'. For the control, β-actin cDNA was amplified with 5'-GAAACTACATTCAATTCC-3' and 5'-ACTCATCGTACTCCTGCT-3' as primers.

### Immunoblotting

Total proteins were extracted with RIPA buffer in presence of protease inhibitors (Roche). Membrane proteins were extracted using Membrane and Cytosol Protein Extraction Kit (Beyotime) according to manufacturer’s instructions. Samples were separated with SDS-PAGE and blotted on a NC membrane (Pall Corporation). Membranes were incubated using these specific primary antibodies: rabbit antibody to RAB18 (1:2000, Sigma), mouse antibody to β-actin (1:5000, Santa Cruz Biotechnology), mouse antibody to FLAG (1:1000, Sigma), mouse antibody to GAPDH (1:4000, Proteintech), rabbit antibody to RAB3GAP2 (1:2000, Novus Biologicals),mouse antibody to N-cadherin (1:1000, BD Transduction Labs), rabbit antibody to NaK ATPase (1:10000, Abcam), rabbit antibody to GRASP65 (1:5000, Abcam), mouse antibody to KDEL (1:1000, Abcam), rabbit antibody to GluR1 (1:2000, Abcam) and mouse antibody to βIII-tubulin (Tuj1) (1:5000, Sigma). We used secondary antibodies to rabbit and mouse HRP. Proteins were revealed using a chemiluminescence kit (Thermo Fisher Scientific).

### In situ hybridization

Nonradioactive RNA in situ hybridization was performed as described previously [23]. In brief, brains from E14.5, E17.5, and P0 mice were fixed overnight at 4 °C with 4 % paraformaldehyde then consecutively dehydrated in 30 % sucrose at 4 °C overnight. Full length cDNA of RAB18 was produced by PCR and cloned into pGEM-T easy vector (Promega) to generate antisense probe for RAB18 according to the supplier instructions. Clones suitable to produce antisense probes labeled with digoxigenin with T7 RNA polymerase (Roche) were selected and probes purified after synthesis using mini Quick Spin RNA columns (Roche). Cryostat sections of 14 μm were cut with a freezing microtome (Leica). The sections were hybridized with probes for 22 h at 63.5 °C. The hybridization signal was detected with anti-DIG antibody coupled to AP (Roche) and staining was obtained with NBT and BCIP (Roche). The images were acquired with OLYMPUS DP72 microscope digital camera system.

### In utero electroporation

Pregnant Institute of Cancer Research (ICR) mice were purchased from Department of Laboratory Animal Science, Peking University Health Science Center. In utero electroporation was performed as described previously [16]. Briefly, E14.5 pregnant mice were deeply anaesthetized with 0.7 % pentobarbital sodium. shRNA plasmids were mixed with pCAGGS vectors expressing GFP at 1:1 ratio (1 μg : 1 μg) with a final concentration of 2 μg μl^−1^; In rescue experiments, shRNA plasmids were mixed with indicated pCAGGS expressing constructs at a 1:3 ratio (1 μg : 3 μg) with a final concentration of 4 μg μl^−1^. shRNA plasmids were mixed with pCAGGS-N-cadherin constructs at a 1:1 ratio with a final concentration of 2 μg μl^−1^. The mixed plasmids solution with 0.05 % Fast Green (Sigma) were injected into the lateral ventricle embryonic brains from outside uterus with a glass micropipette. Immediately after DNA injection, an electrode was positioned flanking the ventricular zones of each embryo and pulsed five times at 33 V for 50 ms with intervals of 1 s, using an ECM 830 electroporator (BTX, Harvard Apparatus, Holliston MA, USA). After electroporation, uterine horns were replaced to the abdominal cavity, allowing embryos to continue developing. Control and experimental embryos were generated from the same litter, and the injections were done on the left and right ventricular zones, respectively, for later recognition.

### Cell culture and transfection and virus infection

HEK293T cells were maintained in DMEM (Invitrogen) supplemented with 10 % fetal bovine serum (Biochrom, Berlin, Germany). Cells were transfected with Lipofectamine 2000 (Invitrogen) according to the manufacturer’s instructions. Brain cortices from embryonic day 16.5 mice were dissected and plated on coverslips coated poly-D-lysine (Sigma). Plasmid vectors were introduced into cortical neurons by transfection with Nucleofector (Amaxa) according to the manufacturer’s instructions. In lentivirus-delivery shRNA, virus was constructed and packed by GeneChem (Shanghai, China), and infection was performed according to the manual.

### Immunostaining

For immunohistochemistry, Coronal sections of 14 μm were cut with a freezing microtome (Leica). The sections were washed in PBS for 3 times, and permeabilized in PBS with 0.4 % Triton X-100 for 30 min at room temperature. The slides were blocked in 5 % bovine serum albumin in PBS for 2 h and were subsequently incubated with primary antibodies overnight at 4 °C. After washing, sections were incubated with the correspondent Alexa-conjugated secondary antibodies (Invitrogen) for 2 h at room temperature. For immunocytochemistry, primary cultured cortical neurons were fixed in 4 % PFA in PBS for 20 min at room temperature. Fixed neurons were permeabilized in PBS with 0.3 % Triton X-100 for 10 min and blocked in 5 % bovine serum albumin in PBS for 2 h at room temperature. Coverslips were stained with indicated primary antibodies overnight at 4 °C followed by correspondent fluorescence-conjugated secondary antibodies for 2 h at room temperature. The images were captured on OLYMPUS confocal microscope or Nikon Eclipse Ti-E inverted microscope with a total internal reflection fluorescence illumination unit.

The following primary antibodies were used: rabbit antibody to RAB18 (1:1000, Sigma), mouse antibody to N-cadherin (1:1000, BD Transduction Labs), rabbit antibody to GFP (1:1000, Molecular Probes), rabbit antibody to Ki67 (1:1000, Abcam), rabbit antibody to Cleaved Caspase-3 (Asp175) (1:1000, Cell Signaling Technology), mouse antibody to Tuj1 (1:5000, Sigma) and mouse antibody to Nestin (1:400, Millipore). Nuclei were visualized with DAPI (1:2000, Sigma). The slides were mounted in Fluoromount-G (Southern Biotechnology Associates).

### Quantitative analysis

All data are presented as the mean ± SEM. Statistical significance was calculated using an unpaired Student’s *t* test, and differences were considered significant at *p* < 0.05. The quantification was performed using the software ImageJ of NIH and Image-Pro Plus 6.0 software (Media Cybernetics). Quantification of neuronal migration was estimated by recording fluorescence intensities of GFP in distinct regions of the cerebral cortices. Fluorescence intensities inside same width rectangles in different regions of the cerebral cortex (CP, IZ, and SVZ/VZ) were measured and relative intensities to the total fluorescence were calculated. Axonal and dendritic length was measured with the ImageJ plug-in NeuronJ 1.2 software. Immunoblots bands analysis were performed using ImageJ. Target proteins and reference protein (Tuj1) bands intensity were measured. We continued to divide the both values by the reference protein value of bands intensity for normalizing the reference protein values to 1 in each group due to the variability in the assay, nevertheless the trend was the same in all experiments. The experiment was performed at least four times.

## Results

### RAB18 is expressed in the developing brain

**Fig. 1:**
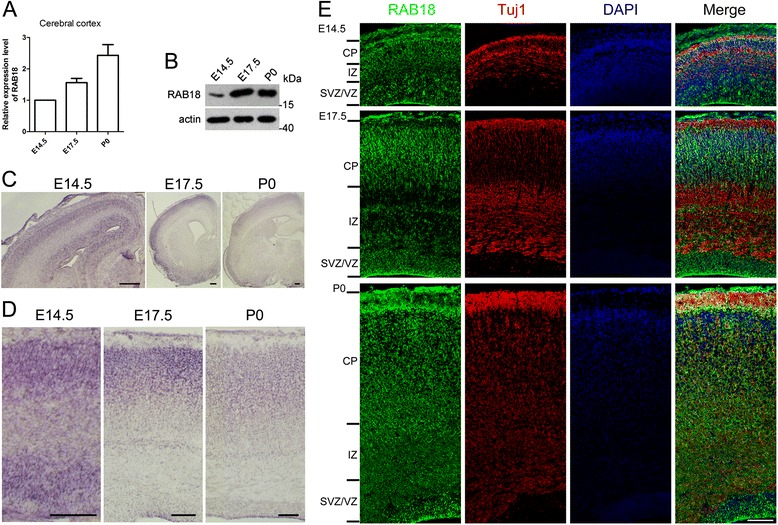
RAB18 is expressed in the developing cerebral cortex. **a** Relative expression levels of RAB18 in cerebral cortex at temporal expression pattern were measured by real time PCR. **b** Immunoblot reveals RAB18 protein levels during the cortical development at the different time points. **c** Expression of RAB18 mRNA was analyzed by in situ hybridization on coronal sections of the telencephalon during development. **d** RAB18 transcript distribution in the cerebral cortex during development. **e** Immunostaining with RAB18 (green), Tuj1 (red) and DAPI (blue) in coronal sections of the E14.5, E17.5 and P0 mouse brain. Scale bars: (**c**), 200 μm; (**d**, **e**), 100 μm. Data are shown as the mean ± SEM

We first investigated the expression pattern of RAB18 in cerebral cortex at different developmental stages in mice. Real-time PCR showed that RAB18 mRNA was expressed in the developing brain and the expression level of RAB18 increased from embryonic day 14.5 (E14.5) to postnatal day 0 (P0) (Fig. 1a). We next measured the level of protein in the developing cerebral cortex, and found the RAB18 expression level increased from E14.5 to P0 (Fig. 1b). Moreover, in situ hybridization indicated that RAB18 mRNA was highly expressed in subventricular and ventricular zones (SVZ/VZ) at E14.5 and expressed in cerebral cortex at E17.5 and P0 (Fig. 1c and d). To confirm that RAB18 is expressed in neurons as indicated by in situ hybridization analysis, intensive RAB18 immunostaining was found in the developing mouse brains. At E14.5, RAB18 was widely expressed in SVZ/VZ. At E17.5 and P0, robust RAB18 protein expression was detected in SVZ/VZ, intermediated zone (IZ) and cortical plate (CP). Together, these data suggest that RAB18 is highly expressed in the developing cortex.

### RAB18 regulates neuronal migration to the cortical plate

**Fig. 2:**
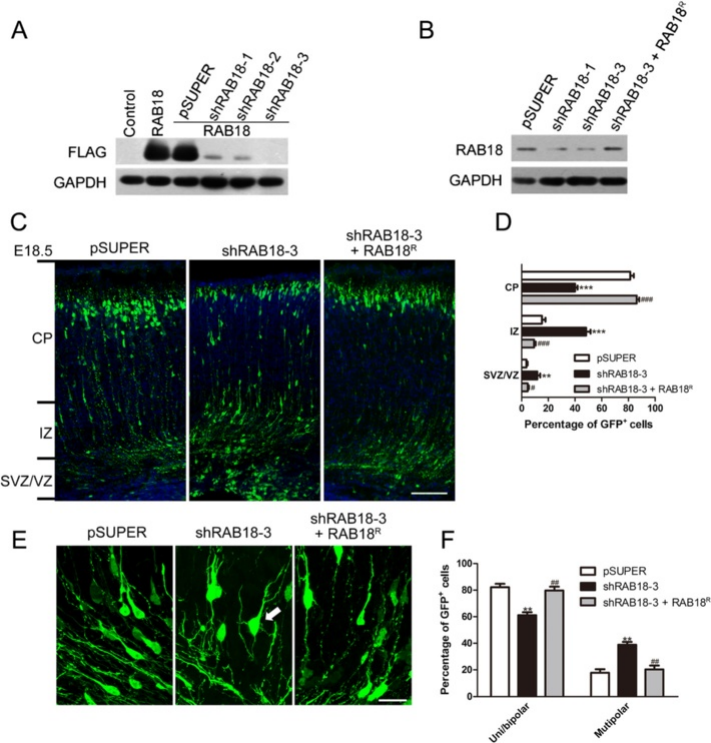
Loss of RAB18 regulates the radial migration of cortical neurons. **a** Immunoblot analysis of FLAG-RAB18 expression in HEK293T cells shows that RAB18 shRNA-1, 2, and 3 (shRAB18-1, shRAB18-2 and shRAB18-3) are efficient to knockdown RAB18. **b** Immunoblot analysis of RAB18 expression in cultured cortical neurons shows that shRAB18-3 is efficient to knockdown endogenous RAB18 and co-transfection with shRAB18-3 and the plasmid expressing RAB18^R^ shows that shRAB18-3 does not apparently reduce RAB18^R^ expression. **c** Representative coronal section showing migration of transfected neurons 4 d after electroporated at E14.5 with GFP plasmid together with indicated constructs. Scale bar: 100 μm. **d** Quantification of percentages of GFP positive neurons in different cortical regions. Data represent the mean ± SEM and the regional distribution of GFP positive neurons is quantitatively analyzed in the cerebral cortex from at least three brains. **e** Magnified images of individual electroporated neurons in intermediate zone. A high percentage of migrating RAB18 knockdown neurons stayed at the multipolar stage (arrowhead). Scale bar: 20 μm. **f** Quantification of the percentages of neurons with uni/bipolar and multipolar morphology. Error bar indicated the SEM of three different brains. ^***^ *p* < 0.001 and ^**^ *p* < 0.01 versus pSUPER; ^###^ *p* < 0.001, ^##^ *p* < 0.01 and ^#^ *p* < 0.05 versus shRAB18-3; *t* test

The expression pattern of RAB18 in mouse brain together with the observed disease phenotype of Warburg Micro syndrome patients led us to hypothesize that RAB18 is important for the cortical development. To test whether RAB18 may regulate neuronal migration, E14.5 mice were transfected with pSUPER and the short hairpin RNA against RAB18 (shRAB18-3) constructs into the developing mouse brain using in utero electroporation. RAB18 knockdown significantly reduced the proportion of neurons reaching the cortical plate (CP) in comparison with pSUPER at E18.5 (Fig. 2a-d). The shRAB18-3 specificity was demonstrated by rescue of neuronal migration following co-electroporation of a plasmid-expressing RAB18 mutant resistant to the shRAB18-3 sequences (RAB18^R^) (Fig. 2b-d) and RAB18 did not apparently affect neuronal migration when it was expressed alone (Additional file 1: Figure S1). Moreover, the ratio of multipolar neurons in RAB18 deficient neurons is substantially higher compared with the control in intermediate zone (IZ) (Fig. 2e and f).

**Fig. 3:**
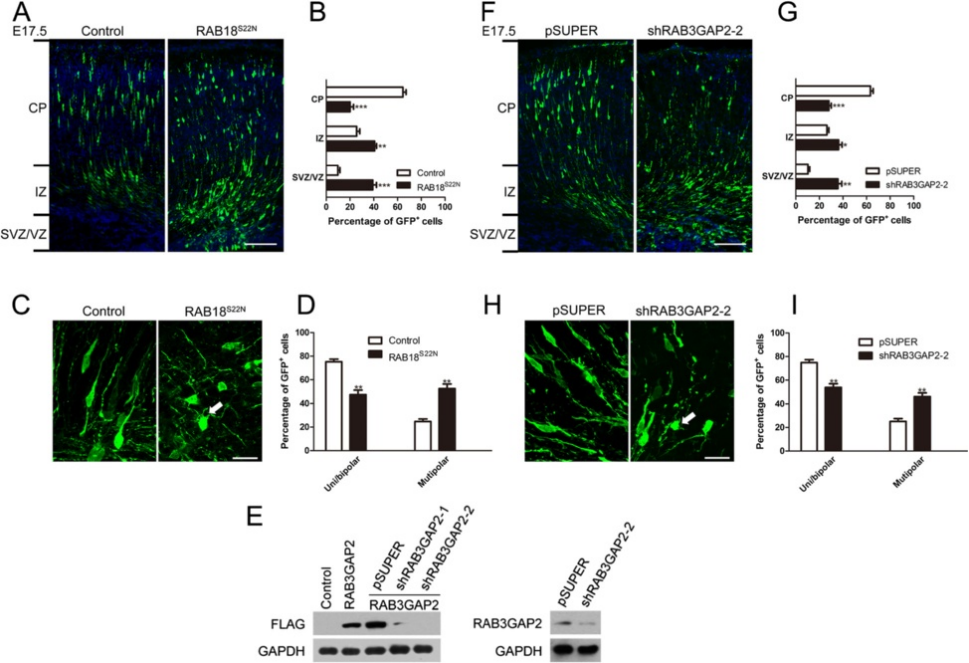
Overexpression of DN-RAB18 (S22N) and suppression of RAB3GAP2 all affect cortical neuronal migration. **a** Representative coronal section showing migration of transfected neurons 3 d after electroporated at E14.5 with GFP plasmid together with indicated constructs. Scale bar: 100 μm. **b** Data represent the mean ± SEM and the regional distribution of GFP positive neurons is quantitatively analyzed in the cerebral cortex from at least three brains. **c** Magnified images of individual electroporated neurons in intermediate zone. A high percentage of migrating DN-RAB18 (S22N) expression neurons stayed at the multipolar stage (arrowhead). Scale bar: 20 μm. **d** Quantification of the percentages of neurons with uni/bipolar and multipolar morphology. Error bar indicated the SEM of three different brains. **e** Immunoblot analysis shows that RAB3GAP2 shRNA-2 (shRAB3GAP2-2) is efficient to knockdown FLAG-RAB3GAP2 and endogenous RAB3GAP2 in HEK293T cells and cultured cortical neurons. **f** Representative coronal section showing migration of transfected neurons 3 d after electroporated at E14.5 with GFP plasmid together with indicated constructs. Scale bar: 100 μm. **g** Quantification of percentages of GFP positive neurons in different cortical regions. Data represent the mean ± SEM and the regional distribution of GFP positive neurons is quantitatively analyzed in the cerebral cortex from at least three brains. **h** Magnified images of individual electroporated neurons in intermediate zone. A high percentage of migrating RAB3GAP2 knockdown neurons stayed at the multipolar stage (arrowhead). Scale bar: 20 μm. **i** Quantification of the percentages of neurons with uni/bipolar and multipolar morphology. Error bar indicated the SEM of three different brains. ^***^ *p* < 0.001, ^**^ *p* < 0.01 and ^*^ *p* < 0.05 versus control (or pSUPER); *t* test

A dominant negative form DN-RAB18 (S22N) is reported artificially engineered inactive GDP-bound Ser22Asn mutant RAB18 [8, 13]. To confirm these results, we electroporated DN-RAB18(S22N) expressing vectors into E14.5 cerebral cortices, and fixed the cortices at E18.5 when a mild impact was detected (Additional file 2: Figure S2). Recent studies suggest that the deficiency of some targeted genes produces more obvious phenotype of inhibition at E17.5 than at E18.5, reminding us to try to observe early [22, 24]. The DN-RAB18 (S22N) expressing vectors were electroporated into E14.5 cerebral cortices, and the cortices were fixed at E17.5. In contrast to control neurons, many DN-RAB18 (S22N) expressing neurons were found stalled near the border between the VZ and IZ (Fig. 3a and b). Some of DN-RAB18 (S22N) expressing neurons had abnormal morphologies, including multiple minor processes (Fig. 3c and d). The RAB3GAP complex originally identified as a cellular factor promoting GTP hydrolysis by RAB3. It consists of two subunits (RAB3GAP1 and RAB3GAP2) [25, 26]. In later work, the function of RAB3GAP1 has been explored and it was found to possess GAP activity to RAB3 independently. However, RAB3GAP2 was characterized as a non-catalytic subunit of the RAB3GAP complex. RAB3GAP2 does not affect the GAP activity of RAB3GAP1 but its function is unclear [5, 25]. Recently, Gerondopoulos et al. demonstrate that RAB3GAP complex is a specific GEF for RAB18. Both of RAB3GAP1 and RAB3GAP2 could be required if RAB3GAP functioned as the RAB18GEF [8]. Base on this point, to further test RAB3GAP mediated activation of RAB18 regulates neuronal migration, we developed shRNAs to RAB3GAP2 which does not affect the function of the GTPase activating protein to RAB3. We observed the position of migrating neurons at E17.5 for 3 d after in utero electroporation of pSUPER as well as RAB3GAP2 shRNA-2 (shRAB3GAP2-2) and results showed that RAB3GAP2 knockdown impaired neuronal migration. The distribution of shRAB3GAP2-2 treated neurons differed significantly from pSUPER (Fig. 3e-g). RAB3GAP2 deficient neurons also developed minor processes (Fig. 3 h and i). At later E18.5 stages, the distributions of neurons do not have apparent differences in pSUPER and RAB3GAP2 knockdown groups (Additional file 3: Figure S3). Together, these results indicate that the activation of RAB18 is critical for radial migration of the cortical neurons.

Loss of RAB18 did not appear to disrupt the nestin labeled radial glial cells which function as a substrate for neuronal migration (Additional file 4: Figure S4A). No significant differences in the staining for proliferative markers Ki67 were found between pSUPER and shRAB18-3 cortices at E16.5 (Additional file 4: Figure S4B and C). The mouse cerebral cortex was labeled with the cell apoptosis marker cleaved caspase-3 and there is no apparent change after silencing RAB18 in neural progenitor cells (Additional file 4: Figure S4D). Thus, our findings establish an important role for RAB18 in neuron radial migration during the cortical development.

### RAB18 regulates cortical neuron morphogenesis in vitro

**Fig. 4:**
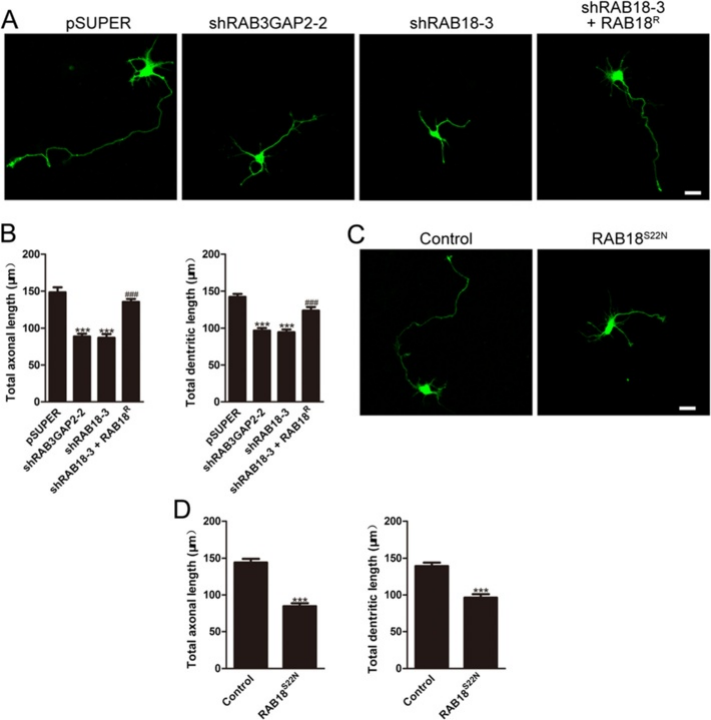
RAB18 regulates neuronal neurite growth in vitro. **a** Representative images of neurons transfected with GFP plasmid together with indicated constructs. Scale bar: 20 μm. **b** Quantitative analysis of total axons and dendrites length of neurons treated as in **a**. Data represent the mean ± SEM and were analyzed by *t* test; ^***^ *p* < 0.001 versus pSUPER; ^###^ *p* < 0.001 versus shRAB18-3; n = 70-90 in each group. **c** Representative images of neurons transfected with plasmids expressing control or DN-RAB18 (S22N) together with GFP plasmid. Scale bar: 20 μm. **d** Quantitative analysis of total axons and dendrites length of neurons treated as in **c**. Data represent the mean ± SEM and were analyzed by *t* test; ^***^ *p* < 0.001 versus control; n = 70-90 in each group

The migratory delay induced by RAB18 deficiency shows the attenuation of neuronal development. This reminded us of the important function of RAB18 in neurite growth during neuronal development. The plasmids encoding GFP together with pSUPER, shRAB3GAP2-2, and shRAB18-3 were transfected into primary cortical neurons respectively. The neurons were allowed to repolarize and were cultured for 3 d. Quantitative analysis demonstrated a difference in the morphology changes of the treated neurons. RAB18 knockdown and disruption of RAB3GAP (RAB18GEF) by RAB3GAP2 knockdown all resulted in a reduction in the total length of both axons and dendrites. These effects were prevented by co-transfecting a shRAB18-3 resistant form of RAB18^R^ (Fig. 4a and b). Moreover, interference with RAB18/RAB18GEF interaction by the overexpression of the DN-RAB18 (S22N) significantly decreased total axons and dendrites length compared with control (Fig. 4c and d). Together, these data indicate that RAB18 regulates cortical neuron morphogenesis in vitro.

### RAB18 knockdown impairs neuron surface N-cadherin

**Fig. 5:**
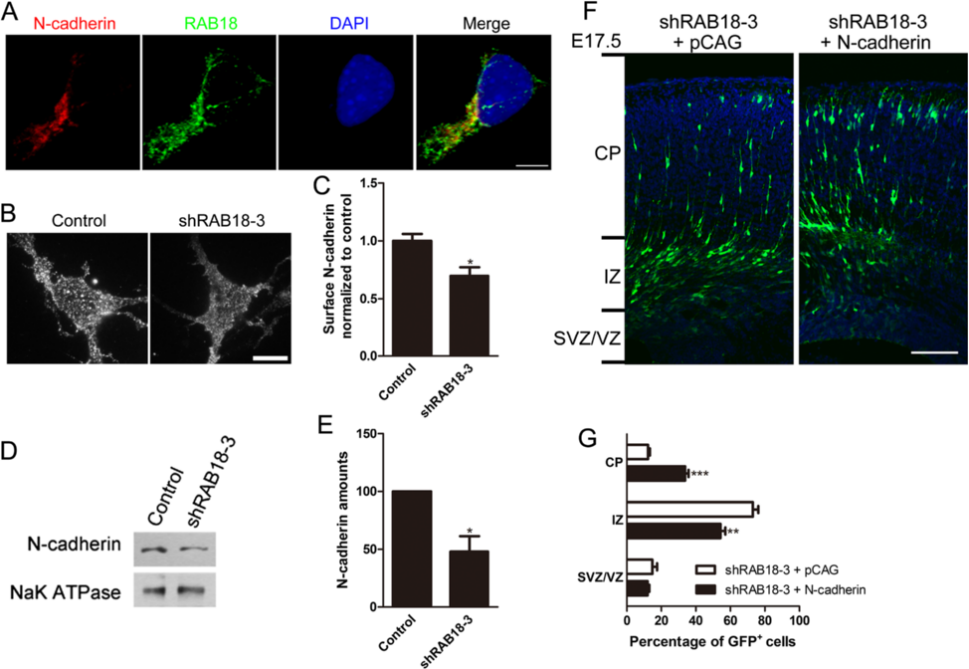
Knockdown of RAB18 impairs the surface expression level of N-cadherin in neuron. **a** Representative images of cortical neurons stained for N-cadherin (red), RAB18 (green) and DAPI (blue). Scale bar: 5 μm. **b** Representative images of surface N-cadherin immunostaining of cortex neurons infected with lentivirus driving the expression of control or shRAB18-3. Scale bar: 5 μm. **c** Quantification of surface N-cadherin from neurons infected with lentivirus expressing control or shRAB18-3 (n = 10, ^*^ *p* < 0.05, *t* test). **d** Immunoblot analysis of membrane proteins of cortical neurons infected with indicated plasmids with the indicated antibodies. **e** The graph indicates the ratio of N-cadherin / NaK ATPase. Knockdown of RAB18 significantly decreased N-cadherin protein level in the membrane of neurons (n = 5, ^*^ *p* < 0.05, *t* test). **f** Representative coronal section showing migration of transfected neurons 3 d after electroporated at E14.5 with GFP plasmid together with indicated constructs (shRAB18-3 + pCAG, n = 4; shRAB18-3 + N-cadherin, n = 4). Scale bar: 100 μm. **g** Quantification of percentages of GFP positive neurons in different cortical regions. Data represent the mean ± SEM and were analyzed by *t* test; ^***^ *p* < 0.001 and ^**^ *p* < 0.01 versus shRAB18-3 + pCAG

In previous studies, RAB5, RAB7 and RAB11 control distinct steps of neuronal migration via the regulation of N-cadherin [16]. We focus on N-cadherin because we found N-cadherin co-localized with RAB18 in neurons (Fig. 5a).

We first analyzed the surface expression level of N-cadherin in RAB18 knockdown neurons by using total internal reflection microscopy which has the advantage to illuminate protein on cell membrane surface. Knockdown of RAB18 resulted in the decrease of N-cadherin on the membrane surface of cultured neurons (Fig. 5b and c). Besides, primary cortical neurons were infected with lentivirus expressing shRAB18-3 or control. We further confirmed that knockdown of RAB18 led to a decrease of the level of N-cadherin on membrane through extracting membrane protein (Fig. 5d and e).

To examine the role of N-cadherin in affecting cortical neurons migration, we introduced shRAB18-3 together with N-cadherin plasmid at E14.5 by in utero electroporation. Previous researches have revealed overexpression of N-cadherin blocks neuronal migration [16, 27]. Mild migration inhibition was observed after overexpression of N-cadherin in our experiment too (Additional file 5: Figure S5). Nevertheless, introduction of moderate N-cadherin in RAB18 knockdown neurons substantially increased the percentage of neurons in CP (Fig. 5f and g). These results suggest that overexpression of N-cadherin could partly rescue the defect of neuronal migration caused by RAB18 knockdown.

### Loss of RAB18 induces an acceleration of N-cadherin degradation

**Fig. 6:**
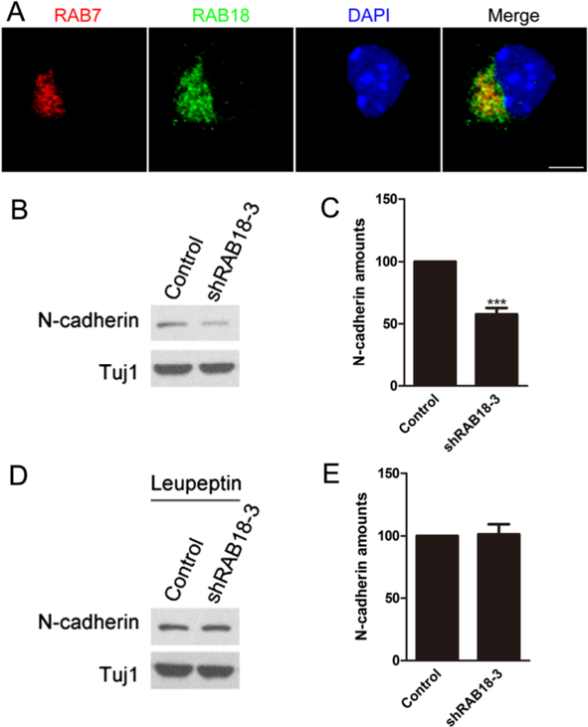
N-cadherin lysosomal degradation is enhanced in RAB18 deficient neurons. **a** Representative images of cortical neurons stained for RAB7 (red), RAB18 (green) and DAPI (blue). Scale bar: 5 μm. **b** Immunoblot analysis of cortical neurons infected with indicated plasmids with the indicated antibodies. **c** The graph indicates the ratio of N-cadherin / Tuj1 in **b**. Knockdown of RAB18 significantly decreased the total N-cadherin protein level of neurons. **d** Immunoblot analysis of cortical neurons infected with indicated plasmids with the indicated antibodies. Leupeptin (50 μM) was done 12 h before harvesting. **e** The graph indicates the ratio of N-cadherin / Tuj1 in **d**. Knockdown of RAB18 has no significant differences in the total N-cadherin protein level of leupeptin treated neurons. Data represent the mean ± SEM and the experiment was performed at least four times. ^***^ *p* < 0.001 versus control

Loss of RAB18 led to the decrease of surface level of N-cadherin on neurons. Previous studies demonstrated that N-cadherin has been identified a target molecule of the RAB7 dependent lysosomal degradation pathway [16]. We first found that RAB18 partially co-localized with RAB7 (Fig. 6a). Next, we found that the knockdown of RAB18 significantly decrease the total expression level of N-cadherin, but the same change did not happen when we treated neurons with leupeptin which can inhibit lysosomal degradation pathway (Fig. 6b-e). RAB18 plays a role in Golgi/endoplasmic reticulum (ER) network which likely affects general mechanisms of protein localization or expression [8, 13]. GRASP65 takes part in the sorting and modification of proteins exported from ER as well as establishing the structure of the Golgi apparatus. The special ER protein containing KDEL motif is necessary to ER localization of plasma membrane protein [28, 29]. We measured their expression by western blot and the results indicated that there is no apparently changed in the total expression of GRASP65 and KDEL after absence of RAB18 (Additional file 6: Figure S6). Besides, previous researches have revealed that the ubiquitin proteasome pathway is essential for the degradation of GluR1 which is not targeted to the lysosomal degradation pathway [30]. No significant changes of GluR1 were found after loss of RAB18 in neurons (Additional file 6: Figure S6). These results clarified that the general mechanisms of protein expression are not apparently affected by RAB18 knockdown. The above data suggest that loss of RAB18 induced an acceleration of N-cadherin degradation resulting in the decrease of surface level of N-cadherin on neurons.

## Discussion

Warburg Micro syndrome is a neurodevelopmental disorder with severe impairment of eye and brain development, including congenital cataract, microcephaly and profound mental retardation [1–7]. RAB18 mutations have been indentified in patients with Warburg Micro syndrome [3]. However, understanding the connection between genotype and disease phenotype is restrained by the lack of information regarding the role of RAB18 in the cortical development. We found that RAB18 is highly expressed in the developing mouse brain. Our finding that RAB18 is essential for neuronal migration provides an important evidence of a cellular mechanism that may explain the development symptoms caused by RAB18 mutations.

**Fig. 7:**
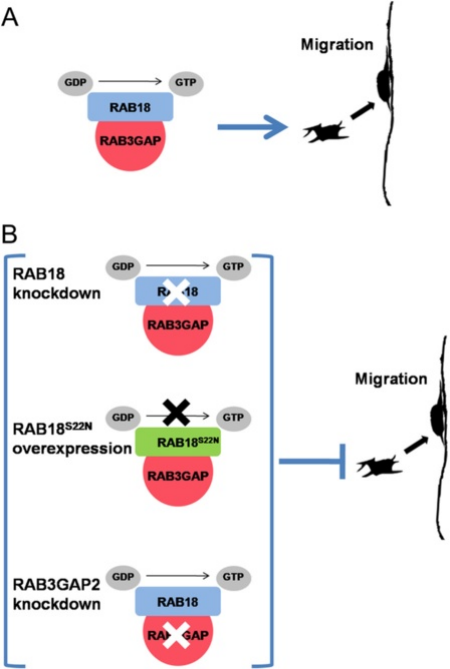
The possible model for the function of RAB18 involved in developing neurons. **a** RAB18-GDP is activated to RAB18-GTP by RAB3GAP (RAB18GEF) in normal neurons. **b** The indicated conditions result in loss of RAB18 or dysregulation of RAB18 activity which impairs neuronal migration

In this study, we discovered a critical function for RAB18 in regulating neuronal migration during brain development. We observed that the reduced number of RAB18 deficient neurons in the CP is not a result of apparent defects in radial glial cells or neuronal proliferation. The disease associated point mutation RAB18 (L24Q) is lack of guanosine nucleotide binding and its variant occurs within the conserved domain, two amino acids downstream of the published dominant negative form DN-RAB18 (S22N) [3]. To confirm the results, we applied overexpression of DN-RAB18 (S22N) and disturbing the RAB3GAP which is identified a GEF for activation of RAB18 [8]. Comparing with RAB18 knockdown which greatly affected neuronal migration, mild migration retardation was observed in DN-RAB18 (S22N) overexpression neurons at E18.5. There may be different mechanisms for these two conditions. In normal neurons, RAB18 with GTP bound form binds and activates specific effector molecules, which induce a downstream cellular response. RAB18 returns to GDP bound form after the effector dissociates upon GTP hydrolysis. Then RAB18-GDP is activated to RAB18-GTP by its GEF (RAB3GAP) and continues this functional cycle in vivo (Fig. 7a). However, the silencing RAB18 makes a direct damage for normal functional cycle of RAB18 because of the disappearance of “substrate”. In contrast, when we overexpressed DN-RAB18 (S22N), large number of RAB3GAP combined with RAB3GAP is in vain, because DN-RAB18 (S22N) has a permanent inactive form which does not fulfill normal function cycle (Fig. 7b). This may be the reason that overexpression of DN-RAB18 (S22N) produces a different migration phenotype with loss of RAB18.

Disruption of RAB3GAP by RAB3GAP2 knockdown obviously produced the impact of neuronal migration at E17.5, however, after which the impact is not obvious at E18.5. For one thing, RAB3GAP2 is a subunit of RAB3GAP complex. RAB3GAP may have residual functions due to only suppression of its part subunit. For another, similar phenomenon was also observed that suppression of GEF for Rac1 has milder phenotype than knockdown of Rac1 in neuronal migration and the reason is that additional GEF of Rac1 was found involving in the process [31]. Warburg Micro syndrome is a devastating developmental disorder with severe brain atrophy. Coincidentally, all patients with RAB3GAP2 mutations have milder brain and clinical phenotype than patients with RAB18 mutations by MRI and clinical observation [7]. These evidences may also imply the distinct phenotype of RAB3GAP2 knockdown in neuronal migration during the cortical development. RAB3GAP is the first GEF of RAB18 reported lately and whether RAB18 has other GEF candidates of compensating function remains to be clarified [8].

It is a complex process for the development of cerebral cortex in which six layered structure is essential for brain function. Proliferating progenitor cells located in the VZ will grow into cortical projection neurons [32, 33]. They display multipolar morphology in the IZ, and transform into bipolar neurons which migrate along radial glial cells with long leading process [34–36]. Several genes such as *LIS1*, *DCX*, *FGF13*, *PIWIL1* and *CDK5*, play crucial roles in neuronal migration and morphological changes through cytoskeletal reorganization, microtubule dynamics and actin reorganization [23, 37–40]. More and more evidence show that RAB family proteins can also take part in radial migration, indicating that membrane trafficking and intercellular signaling play an important role in the development of cerebral cortex [16]. Our data have revealed that neuronal migration is regulated by RAB3GAP mediated activation of RAB18 pathway and support physiological importance of RABs dependent pathways.

N-cadherin regulates neuronal migration in the developing cortex by mediating the interaction between migration neurons and radial glial cells, and changes in the N-cadherin expression level result in neuronal migration defect [16, 27]. Our results showed that loss of RAB18 decreases the level of N-cadherin in neurons but it is not clear exactly whether RAB18 directly regulates N-cadherin or not. N-cadherin can be sorted through many trafficking pathways, including lysosome degradation, recycling to recycling endosome, retrograde transport to Golgi/ER network as well as late endosome/Golgi [16]. The trafficking of N-cadherin may be involved in RAB18 which plays an important role in membrane trafficking [8, 9, 11–13]. We suspect that loss of RAB18 increase the trafficking of N-cadherin through lysosome for degradation. Besides, our results found that N-cadherin overexpression can affect neuronal migration and partly rescue the migration deficiency caused by loss of RAB18. These results support previous studies that N-cadherin is necessary for neuronal migration and overexpression or loss of N-cadherin inhibits migration [16, 27]. Moreover, some studies have also revealed that overexpression of N-cadherin can rescue migration inhibition in knockdown of targeted gene decreasing N-cadherin expression [24, 27].

Two knockout animal models for RAB18 have been described lately. Carpanini et al. reported the characterization of *RAB18*^-/-^ mouse model similar to the Warburg Micro syndrome phenotype and found that loss of RAB18 leads to widespread disruption of neural cytoskeleton in peripheral nerve which provides the possible role of RAB18 into molecular mechanism in some parts of peripheral nervous system [41]. Later, Cheng et al. observed that the N-ethyl-N-nitrosourea-induced *RAB18*^-/-^ mice have the thinner corpus callosum which harbors axonal projections predominantly derived from upper layer neurons suggesting that hypoplastic corpus callosum may be due to defective neurodevelopment and closely related with retardant neuronal migration at prenatal time [21, 22]. Moreover, a translational convergent functional genomics (CFG) approach has identified RAB18 as a candidate gene involved in schizophrenia [42]. Primarily, our results show that RAB18 is a key regulator during the development of brain in mouse, and may demonstrate an essential role of this gene in pathophysiology of Warburg Micro syndrome in humans. Some candidate effectors of RAB18 have been found recently [43]. It remains to be determined how RAB18 dysfunction contributes to disease pathology in detail.

## Conclusions

In summary, our results reveal that RAB18 is expressed in the developing cerebral cortex and affects neuronal migration. This study discovers a novel function of RAB3GAP-RAB18 pathway in the cortical development. It also provides the first evidence of a cellular mechanism that may account for the developmental effects caused by RAB18 mutations and the disease phenotypes (microcephaly, thin corpus callosum and profound mental retardation) observed in patients with Warburg Micro syndrome.

## Additional files

Additional file 1: Figure S1. There is no apparently changed after overexpression of RAB18 in cortical neuronal migration. **A** Representative coronal section showing migration of transfected neurons 4 d after electroporated at E14.5 with GFP plasmid together with indicated constructs. Scale bar: 100 μm. **B** Quantitative analysis of GFP positive neurons in different cortical regions in **A**. Data represent the mean ± SEM (n = 3). (PDF 76 kb) Additional file 2: Figure S2. Overexpression of DN-RAB18 (S22N) mildly impairs the neuronal migration at E18.5. **A** Representative coronal section showing migration of transfected neurons 4 d after electroporated at E14.5 with GFP plasmid together with indicated constructs. Scale bar: 100 μm. **B** Quantitative analysis of GFP positive neurons in different cortical regions in **A**. Data represent the mean ± SEM (n = 3); ^*^ *p* < 0.05 versus control; *t* test. (PDF 78 kb) Additional file 3: Figure S3. The distributions of neurons do not have apparent differences after suppression of RAB3GAP2 at E18.5. **A** Representative coronal section showing migration of transfected neurons 4 d after electroporated at E14.5 with GFP plasmid together with indicated constructs. Scale bar: 100 μm. **B** Quantitative analysis of GFP positive neurons in different cortical regions in **A**. Data represent the mean ± SEM (n = 3). (PDF 70 kb) Additional file 4: Figure S4. There is no apparently changed after silencing RAB18 in radial glial organization, progenitor proliferation and survival. **A**, **B** Cerebral cortical sections at E18.5 and E16.5 which were electroporated at E14.5 with the indicated constructs together with GFP plasmid, were immunostained with antibodies against nestin or Ki67. Scale bars: (**A**), 100 μm; (**B**), 40 μm. **C** Quantification of the ratio of Ki67 and GFP double positive cells to total GFP positive cells in SVZ/VZ (Ki67 index). No significant differences between pSUPER and shRAB18-3 groups are found. Data are shown as the mean ± SEM (n = 3). **D** Immunostaining for cleaved caspase-3 (Asp175) shows no detectable apoptotic cell in RAB18 silencing brains at E16.5 which were electroporated at E14.5. Scale bars: 40 μm. (PDF 210 kb) Additional file 5: Figure S5. Cortical neuronal migration is mildly affected by overexpression of N-cadherin. **A** Representative coronal section showing migration of transfected neurons 3 d after electroporated at E14.5 with GFP plasmid together with indicated constructs. Scale bar: 100 μm. **B** Quantitative analysis of GFP positive neurons in different cortical regions in **A**. Data represent the mean ± SEM (n = 3); ^**^ *p* < 0.01 versus control; *t* test. (PDF 75 kb) Additional file 6: Figure S6. There is no apparently changed after RAB18 deficiency in GluR1, KDEL and GRASP65 protein expression levels. **A** Immunoblot analysis of cortical neurons infected with indicated plasmids with the indicated antibodies. **B**-**D** The graphs separately indicate the ratios of GluR1 / Tuj1, KDEL / Tuj1, and GRASP65 / Tuj1 in **A**. Data represent the mean ± SEM and the experiment was performed at least four times. (PDF 57 kb)

## Abbreviations

RAB3GAP1: RAB3 GTPase activating protein subunit 1
RAB3GAP2: RAB3 GTPase activating protein subunit 2
TBC1D20: TBC1 domain family member 20
GEF: Guanine nucleotide exchange factor
GAP: GTPase activating protein
VZ: Ventricular zone
SVZ: Subventricular zone
CP: Cortical plate
IZ: Intermediate zone
DN: Dominant negative form
LIS1: Lissencephaly type 1
DCX: Doublecortin
FGF13: Fibroblast growth factor 13
PIWIL1: PIWI-like 1
CDK5: Cyclin-dependent kinase 5
CFG: Convergent functional genomics
GAPDH: Glyceraldehyde-3-phosphate dehydrogenase
HRP: Horseradish peroxidase
