# Supplementary figures and images for: RAB18, a protein associated with Warburg Micro syndrome, controls neuronal migration in the developing cerebral cortex
Source: Mol Brain. 2016 Feb 16;9:19. doi: 10.1186/s13041-016-0198-2 (PMC4754921; doi:10.1186/s13041-016-0198-2)

**A**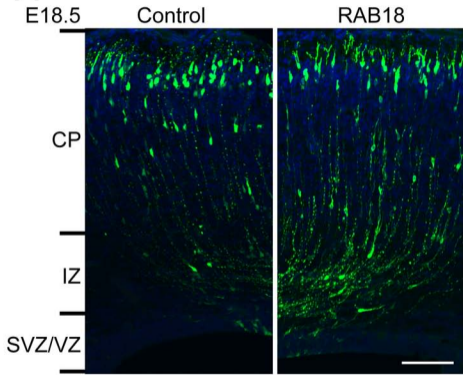**B**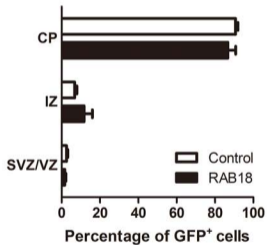

Supplement: Additional file 1: Figure S1. — There is no apparently changed after overexpression of RAB18 in cortical neuronal migration. A Representative coronal section showing migration of transfected neurons 4 d after electroporated at E14.5 with GFP plasmid together with indicated constructs. Scale bar: 100 μm. B Quantitative analysis of GFP positive neurons in different cortical regions in A. Data represent the mean ± SEM (n = 3). (PDF 76 kb) [file 13041_2016_198_MOESM1_ESM.pdf]

**A**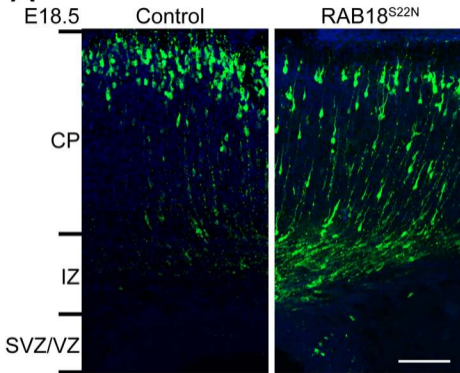**B**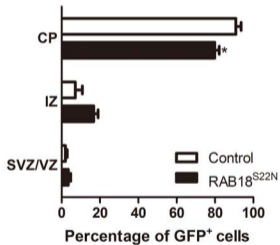

Supplement: Additional file 2: Figure S2. — Overexpression of DN-RAB18 (S22N) mildly impairs the neuronal migration at E18.5. A Representative coronal section showing migration of transfected neurons 4 d after electroporated at E14.5 with GFP plasmid together with indicated constructs. Scale bar: 100 μm. B Quantitative analysis of GFP positive neurons in different cortical regions in A. Data represent the mean ± SEM (n = 3); * p < 0.05 versus control; t test. (PDF 78 kb) [file 13041_2016_198_MOESM2_ESM.pdf]

**A**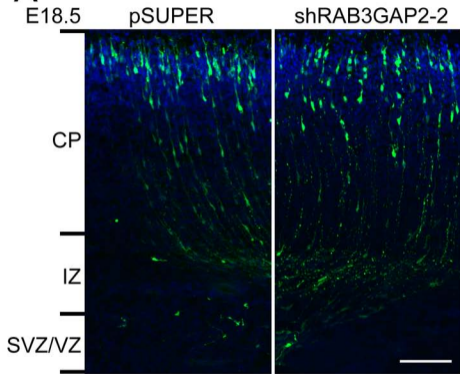**B**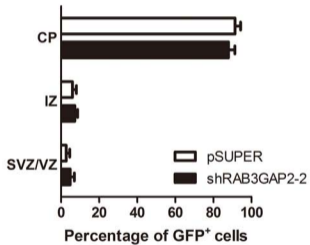

Supplement: Additional file 3: Figure S3. — The distributions of neurons do not have apparent differences after suppression of RAB3GAP2 at E18.5. A Representative coronal section showing migration of transfected neurons 4 d after electroporated at E14.5 with GFP plasmid together with indicated constructs. Scale bar: 100 μm. B Quantitative analysis of GFP positive neurons in different cortical regions in A. Data represent the mean ± SEM (n = 3). (PDF 70 kb) [file 13041_2016_198_MOESM3_ESM.pdf]

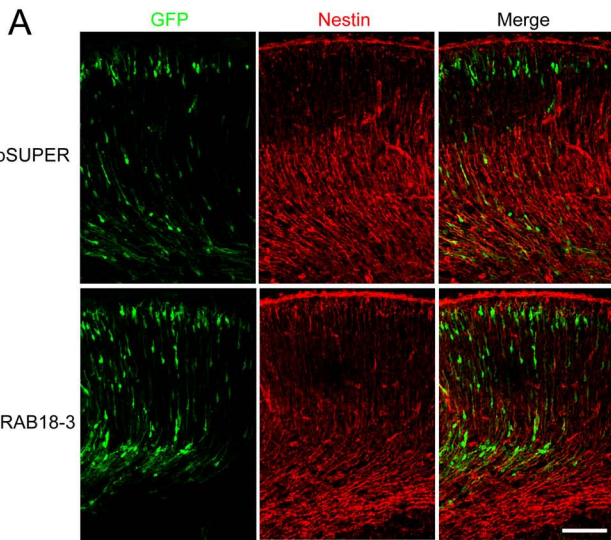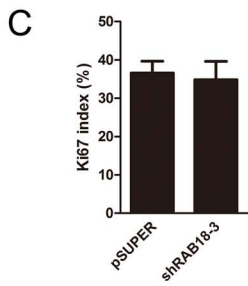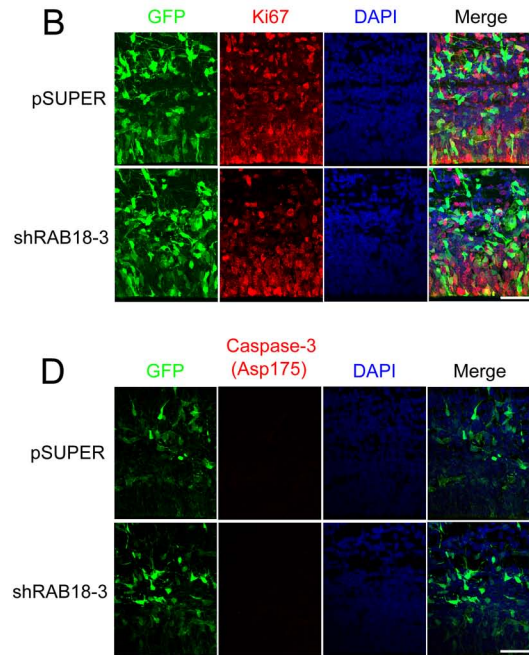

Supplement: Additional file 4: Figure S4. — There is no apparently changed after silencing RAB18 in radial glial organization, progenitor proliferation and survival. A, B Cerebral cortical sections at E18.5 and E16.5 which were electroporated at E14.5 with the indicated constructs together with GFP plasmid, were immunostained with antibodies against nestin or Ki67. Scale bars: (A), 100 μm; (B), 40 μm. C Quantification of the ratio of Ki67 and GFP double positive cells to total GFP positive cells in SVZ/VZ (Ki67 index). No significant differences between pSUPER and shRAB18-3 groups are found. Data are shown as the mean ± SEM (n = 3). D Immunostaining for cleaved caspase-3 (Asp175) shows no detectable apoptotic cell in RAB18 silencing brains at E16.5 which were electroporated at E14.5. Scale bars: 40 μm. (PDF 210 kb) [file 13041_2016_198_MOESM4_ESM.pdf]

**A**

E17.5

Control

N-cadherin

CP

IZ

SVZ/VZ

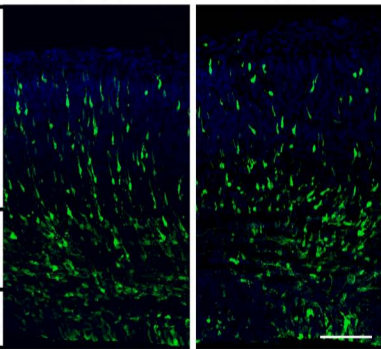**B**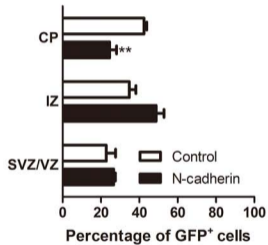

Supplement: Additional file 5: Figure S5. — Cortical neuronal migration is mildly affected by overexpression of N-cadherin. A Representative coronal section showing migration of transfected neurons 3 d after electroporated at E14.5 with GFP plasmid together with indicated constructs. Scale bar: 100 μm. B Quantitative analysis of GFP positive neurons in different cortical regions in A. Data represent the mean ± SEM (n = 3); ** p < 0.01 versus control; t test. (PDF 75 kb) [file 13041_2016_198_MOESM5_ESM.pdf]

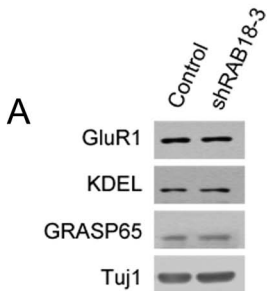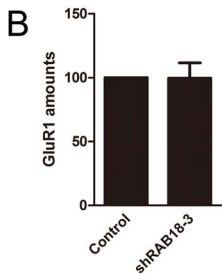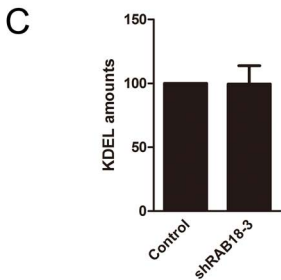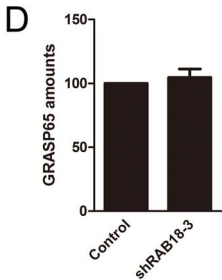

Supplement: Additional file 6: Figure S6. — There is no apparently changed after RAB18 deficiency in GluR1, KDEL and GRASP65 protein expression levels. A Immunoblot analysis of cortical neurons infected with indicated plasmids with the indicated antibodies. B-D The graphs separately indicate the ratios of GluR1 / Tuj1, KDEL / Tuj1, and GRASP65 / Tuj1 in A. Data represent the mean ± SEM and the experiment was performed at least four times. (PDF 57 kb) [file 13041_2016_198_MOESM6_ESM.pdf]
